# Reverse Scan Conversion and Efficient Deep Learning Network Architecture for Ultrasound Imaging on a Mobile Device

**DOI:** 10.3390/s21082629

**Published:** 2021-04-08

**Authors:** Kunkyu Lee, Min Kim, Changhyun Lim, Tai-Kyong Song

**Affiliations:** 1 Department of Electronic Engineering, Sogang University, Seoul 04107, Korea; gklee77@sogang.ac.kr; 2 Hansono, Seoul 08590, Korea; drxmin@hansono.com (M.K.); limchl12@naver.com (C.L.)

**Keywords:** point-of-care, on-device AI, portable ultrasound, mobile system, classification

## Abstract

Point-of-care ultrasound (POCUS), realized by recent developments in portable ultrasound imaging systems for prompt diagnosis and treatment, has become a major tool in accidents or emergencies. Concomitantly, the number of untrained/unskilled staff not familiar with the operation of the ultrasound system for diagnosis is increasing. By providing an imaging guide to assist clinical decisions and support diagnosis, the risk brought by inexperienced users can be managed. Recently, deep learning has been employed to guide users in ultrasound scanning and diagnosis. However, in a cloud-based ultrasonic artificial intelligence system, the use of POCUS is limited due to information security, network integrity, and significant energy consumption. To address this, we propose (1) a structure that simultaneously provides ultrasound imaging and a mobile device-based ultrasound image guide using deep learning, and (2) a reverse scan conversion (RSC) method for building an ultrasound training dataset to increase the accuracy of the deep learning model. Experimental results show that the proposed structure can achieve ultrasound imaging and deep learning simultaneously at a maximum rate of 42.9 frames per second, and that the RSC method improves the image classification accuracy by more than 3%.

## 1. Introduction

Point-of-care ultrasound (POCUS) is an efficient tool for providing diagnostic imaging at the time and place of patient care [1]. Recently, POCUS has become more convenient to use with mobile device-based ultrasound scanners such as Healcerion, Butterfly iQ (Butterfly Network Inc.), and Clarius (Clarius Mobile Health Corp.). These devices have contributed to the expansion of POCUS applications to deliver novel clinical benefits to patients [2].

With the development of this new trend, the use of ultrasound devices by unskilled and non-medical staff has become widespread [3]. As ultrasound imaging is generally performed to obtain diagnostic information in real time, POCUS users must be properly trained or technically supported at the time of ultrasound scanning. This is achievable using computer-aided diagnosis tools.

The most in-demand technical support for unskilled POCUS users might be imaging guidance, providing information on what organs are shown in each image frame [4] and whether or not the target organs are observed in the correct scan plane [5]. Automatic organ classification is essential for realizing such an imaging guidance function. Notably, object classification is also required for other advanced functions, such as automatic diagnosis or measurement of important diagnostic metrics.

Convolutional neural networks (CNNs), which made major progress in recent complex machine vision problems, have been reported to surpass human accuracy [6] in applications such as classification [7], segmentation, and object detection [8,9]. Thus, CNNs have been widely used for the classification of abdominal ultrasound images [10] and for landmark detection of organs [11]. Thus, CNN might be the most suitable method for developing the technology for imaging guidance in POCUS.

There are two ways to implement deep learning network (DLN) inference for object classification using mobile device-based ultrasound scanners. In cloud-based inference, ultrasound images are sent to a cloud web server to compute the labels, and the results are transferred back to the mobile device. In contrast, in on-device artificial intelligence (AI), DLN inference is fully performed on a mobile device without network access [12]. Until recently, some studies have recommended the cloud computing approach using Amazon Web Services (AWS) or Azure for fast real-time implementation of CNN-based object classification, and large datasets to be easily ingested and managed to train algorithms [13,14].

However, cloud computing is not adequate for POCUS with mobile device-based ultrasound scanners, particularly when and where fast internet and wireless communication networks are not available, such as in remote areas and underdeveloped countries with poor communication infrastructure [15]. Moreover, cloud computing is vulnerable as it may attempt to violate the protection of personal information [16].

Therefore, it is advisable to implement deep learning on mobile devices without accessing the Internet. Recently, owing to rapid advances in high-performance parallel computing architecture, mobile devices are already capable of real-time software implementation of DLN using a mobile graphics processing unit (GPU). Accordingly, numerous efforts have been made to implement on-device inference using edge computing [17]. Hitherto, some efficient networks that reduce the calculation quantity and amount of memory for real-time DLN inference on a mobile device have likewise been developed [18,19].

However, in mobile device-based ultrasound scanners, entire back-end signal/image processing tasks to reconstruct images at a typical speed of 20–40 frame per second (fps) must be carried out with the same GPU. This is a major limiting factor for high-speed DLN inference in a mobile device, and to the best of our knowledge, only a few companies have developed simultaneous implementation of ultrasound back-end signal/image processing and DLN for real-time ultrasound medical imaging [20].

**Figure 1:**
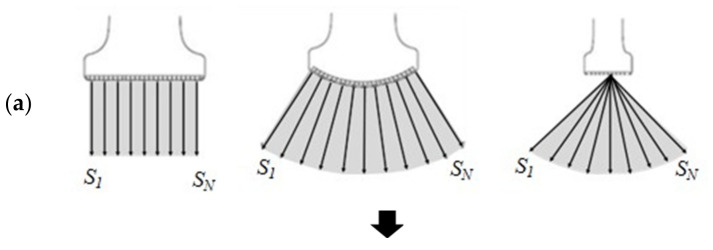

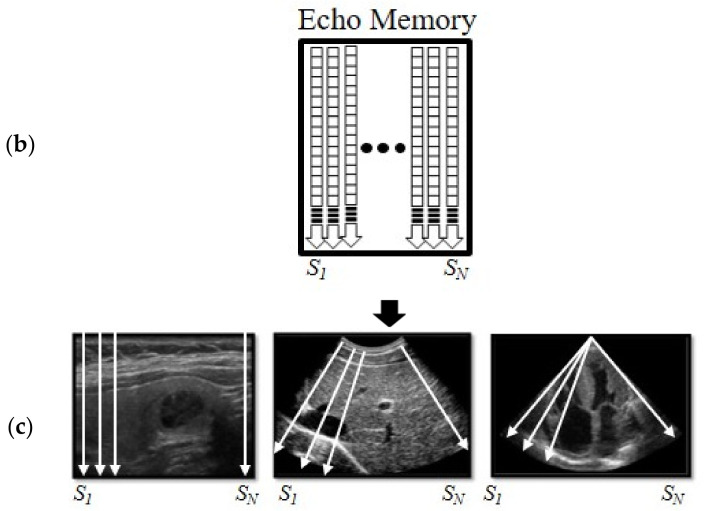
(**a**) *N* scanlines made of three transducers (left panel: linear array, center panel: convex array, right panel: sector phased array.) (**b**) Echo memory is stored to *#scanlines* by *#samples*, regardless of type of transducers. (**c**) *I_USI_*, digital scan-converted is reconstructed as an image with spatial information.

Notably, in ultrasound imaging, the accuracy of the DLN inference is affected by the shape of the field of view (FOV) [21]. Figure 1a shows the three most widely used types of ultrasound array transducers, namely, linear, curved linear (or convex), and sector phased arrays, where the solid arrow lines ($S_{1}\sim S_{N}$) represent the scan lines forming a single image frame [22]. Echo signals along all scan lines, which are obtained after transmission, reception, and beamforming operations along each scan direction, are stored in the echo memory in the same format as shown in Figure 1b. Image formation is achieved by organizing the lines of echo memory and processing them through a digital scan converter (DSC) that transforms them into a raster scan format for display on a video or personal computer (PC) monitor (see Figure 1c) [23].

**Figure 2:**
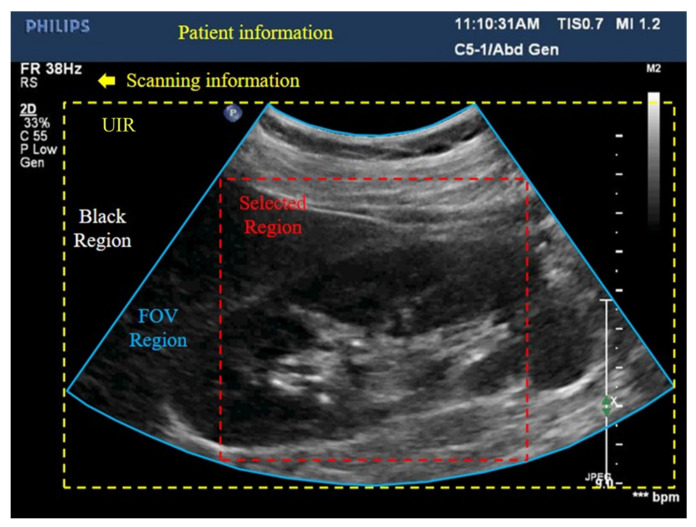
Digital scan-converted ultrasound image (kidney) shown on display. This ultrasound image (USI) region includes not only the field of view (FOV) region (blue box), but also the black region according to FOV and various information. To train ultrasound images, they are generally cropped to an USI region (yellow dotted rectangle) or selected region (red dotted rectangle).

The resulting ultrasound images are displayed on the ultrasound image region (UIR), in a fixed rectangular area (yellow box in Figure 2) on a monitor screen. When the entire UIR is used as training data, not only echo information, but the FOV shape can be recognized as a feature, thus lowering the classification accuracy [21]. To avoid this, a selected region (red box in Figure 2) can be cropped within each FOV as training data. In this case, however, some objects can be partially cut off, which is another factor that lowers the classification accuracy.

**Figure 3:**
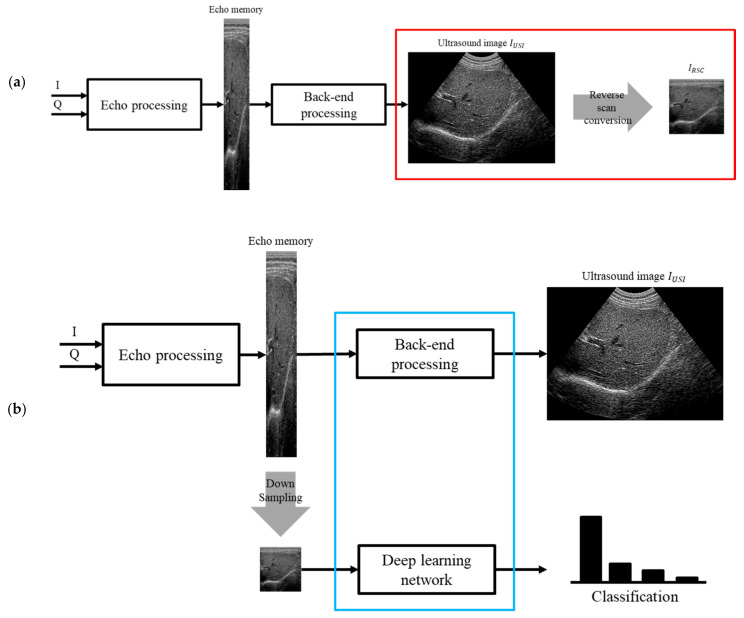
(**a**) Ultrasound image formation processing structure and reverse scan conversion training data building procedure using *I_USI_*, echo memory data (red box). (**b**) For real-time ultrasound imaging and inference, we propose a blue box structure that can effectively compute back-end processing and the deep learning network simultaneously.

We propose an ultrasound AI edge-computing method for mobile device-based ultrasound scanners that perform ultrasound image reconstruction and DLN inference for object classification at a high frame rate required for practical ultrasound diagnosis. We propose to use a pre-DSC image, such that the entire uncropped image is used for training. In this case, all objects in the pre-DSC image are geometrically distorted. In this approach, the DLN was trained to accurately classify the target objects in distorted images. However, no commercial ultrasound scanners provide such pre-DSC images. We refer to this procedure of obtaining pre-DSC images from DSC images as the reverse scan conversion (RSC). Figure 3a illustrates the proposed method for building pre-DSC images. We collect back-end processed *I_USI_* and subsequently generate *I_RSC_* using reverse-scan conversion to train the DLN.

In contrast, in inference (see Figure 3b), echo memory data are down-sampled and used as input to the DLN. If the DLN is implemented after back-end processing on a mobile device, it has a low ultrasonic imaging frame rate. To overcome this limitation, we propose a structure where the DLN is divided to implement ultrasound image reconstruction and DLN inference in real time. The proposed method is evaluated on a mobile device with a portable ultrasound system [3,24], and DLN models are implemented by transferring network coefficients trained in a PC workstation to a mobile device.

## 2. Methods

### 2.1. Reverse Scan Conversion

In diagnostic ultrasound imaging, it is customary to use a specific array transducer for a particular examination, such as a phased array for echocardiography, a convex array for the abdomen, and a linear array for the thyroid [25]. Therefore, if such conventional images are used as a training dataset, the CNN can learn the geometry of the FOV as a feature. This would negatively affect the accuracy of inference when the trained CNN is used to classify a particular organ with an input image obtained by using array transducers other than the one specific to it. This can also occur in POCUS using a mobile device-based ultrasound scanner that is usually equipped with only a one-array transducer and provides a fixed FOV shape.

Another problem with DLN inference in a mobile device-based ultrasound scanner is that high frame rate imaging may be hindered if DLN inference is followed by ultrasound back-end signal/image processing. To solve this problem, DLN inference must be possible without compromising the imaging frame rate by performing both procedures with all GPU resources. As described in the previous section, this problem can be easily solved by using the pre-DSC image data as a training dataset.

**Figure 4:**
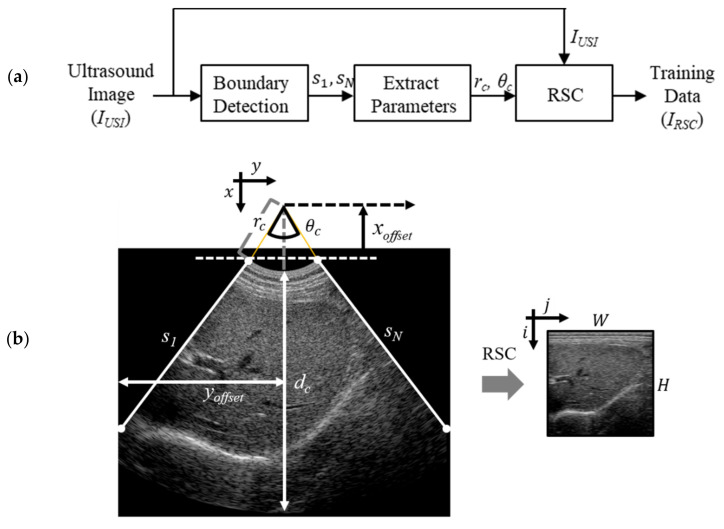
(**a**) Pre-processing procedure for the training data in the ultrasound dataset. *r_c_* and *θ_c_* are extracted from lines *s*_1_ and *s_N_*. (**b**) Coordinate transforming a scan-converted image (*I_USI_*) to RSC image (*I_RSC_*). Left boundary, first scanline, between FOV and black region, is called *s*_1_, whereas the right boundary, last scanline, is referred to as *s_N_*. *i* and *j* are coordinate axes in *I_RSC_*. *I_USI_* has *x* and *y* coordinate axes.

In this study, we propose an RSC method to restore the pre-DSC data from conventionally acquired and labeled images (*I_USI_*) that have been used in previous DLN studies. Figure 4 shows the processing steps used to obtain pre-DSC data.

In the first step, all the scanning and patient information (see Figure 2) are removed, and the UIR is converted into a binary image. Then, by detecting boundaries in the binarized UIR, the left and right FOV edges are found in the binary image to separate the FOV and black regions. Subsequently, *N* scanlines (*s*_1_~*s_N_*) are allocated over the FOV with a uniform angle distribution: *s*_1_ represents the first scanline (i.e., left boundary) and *s_N_* the *N_th_* scanline (right boundary), as illustrated in Figure 4b for the convex array case.

In the second step of Figure 4a, the parameters to express the polar coordinates of the FOV in Figure 4b were extracted using the Hough transformation [26]. The extracted parameters include the transducer radius (*r_c_*), FOV angle (*θ_c_*), *x_offset_*, and *y_offset_*.

Finally, for the pre-DSC image, $I_{RSC}\left(i,\;j\right)$ is obtained in polar coordinates by applying RSC to $I_{USI}\left(x,\;y\right)$ in Cartesian coordinates as follows:

(1) $$I_{RSC}\left(i,\;j\right)=B\left(T(I_{USI}\left(x,\;y\right))\right),$$

where T and B represent the coordinate transformation and bilinear interpolation, respectively. Here, the coordinate transform, T, is determined according to the following relationship:

(2) $$x=\left(r_{c}+i∆r\right)\times \cos\left(-\theta_{c}/\;2+j∆\theta \right)+x_{offset},\;y=\left(r_{c}+i∆r\right)\times \sin\left(-\theta_{c}/\;2+j∆\theta \right)+y_{offset},$$

where $∆r=d_{c}/\;H$ and $∆\theta =\theta_{c}/\;W$, where *W* and *H* denote the width and height of the *I_RSC_*, respectively. Equation (2) for i and j is expressed as follows:

(3) $$i=\frac{1}{∆\theta }\left(\arctan\left(\frac{y-y_{offset}}{x-x_{offset}}\right)+\frac{\theta_{c}}{2}\right),\;j=\;\frac{\sqrt{{\left(x-x_{offset}\right)}^{2}+{\left(y-y_{offset}\right)}^{2}}-r_{c}}{∆r}$$

There are pixels (i.e., holes) that are not assigned to appropriate pixel values. Notably, the bilinear interpolation, B, is employed, such that those holes must be assigned appropriate values at $I_{RSC}\left(i,\;j\right)$.

### 2.2. Structure on a Mobile Device–Frame Asynchronous Classification (FAC)

**Figure 5:**
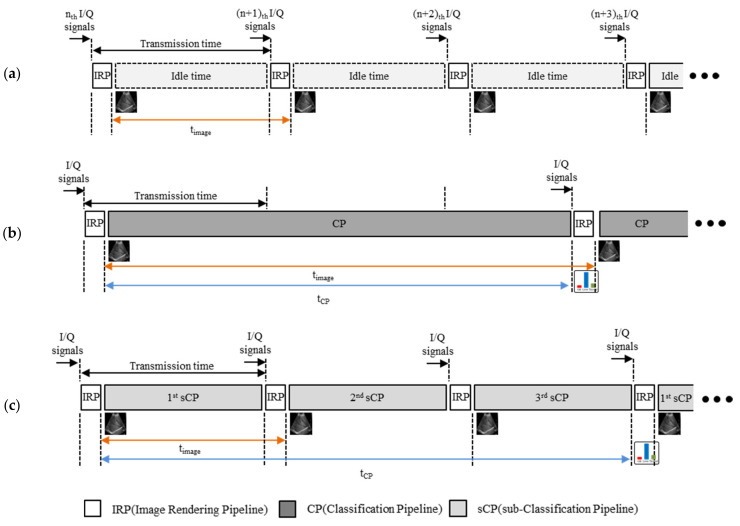
(**a**) Ultrasound image reconstruction. After image rendering pipeline (IRP) for imaging, the system must wait to receive subsequent signals due to the data transfer time. After each IRP, the ultrasound image is displayed on a mobile phone. The time period to form each ultrasound image frame is denoted by the orange bidirectional arrow. (**b**) frame synchronous classification (FSC) structure of a classification through deep learning with ultrasound image reconstruction in a mobile device. Classification time is denoted by the blue bidirectional arrow. (**c**) For real-time processing, a frame asynchronous classification (FAC) structure is made of IRP and sub-classification pipelines (sCPs).

The ultrasonic signals transferred from a portable ultrasound scanner to a mobile device are in-phase/quadrature (IQ) signals. First, echo processing is applied to the IQ signals, which generates the echo memory data. Echo processing is a chain of signal processing functions, such as envelope detection and log compression. Then, the echo memory data is fed to the back-end processing block that includes the DSC. Finally, the DSC output ($I_{USI}$) is displayed on the mobile device. As shown in Figure 5a, the time period to form each image frame (*t_image_*) is not determined by the processing time of the IRP (*t_IRP_*). This is because the portable ultrasound device takes time to transfer ultrasonic signals to the mobile device, and *t_IRP_* is very short compared to the data transfer time. Therefore, the (*n* + 1)*_th_* frame cannot be reconstructed immediately after the *n_th_* frame is reconstructed—there is an idle time during which the IQ signals of the (*n* + 1)*_th_* frame are being transferred.

To add a classification network in this structure, we can add a classification pipeline (CP) that implements a classification network after IRP, as shown in Figure 5b. In this structure, to implement the ultrasound image reconstruction and the classification network in real time, the entire CP must be completed within the idle time. However, the CP processing time is significantly longer than the idle time. In Figure 5b, because the entire CP is processed after the IRP, the time period to form each image frame (see orange line) is increased as much as the CP processing time (see blue line), which eventually takes longer than the data transfer time. Therefore, the ultrasound image reconstruction for the next frame is delayed. We call this the frame synchronous classification (FSC) structure [27], which has the disadvantage of delaying *t_image_* as much as the classification processing time (*t_CP_*).

To overcome this disadvantage of the FSC structure, we propose a structure that can implement ultrasound image reconstruction and a classification network in real time on a mobile device, as shown in Figure 5c. The key purpose of the proposed structure is to split the CP into several sub-pipelines to obtain sub-classification pipelines (sCPs) for an idle time that does not exceed the data transfer time. This structure is called frame asynchronous classification (FAC), as multiple frames are displayed during one classification result. The *t_CP_* of the FAC structure is slightly longer than that of the FSC structure due to the IRPs between the sCPs. However, in real time, because the frame rate is more important than the classification rate, it does not matter whether *t_CP_* is slightly longer.

Further, when the CP takes a long time to process, it can be split into a large number of sCPs. This is because *t_IRP_* is very short compared to the processing time of sCPs (*t_sCP_*), as shown in Figure 5b. Therefore, even if the CP is divided into several sCPs to process ultrasound image reconstruction and DLN inference, the classification rate ($f_{CP}$) has little loss. Let *M* denote the number of times the CP is divided into idle time, then

(4) $$f_{image}\approx M\times f_{CP}$$

where $f_{image}=1/\left(t_{IRP}+t_{sCP}\right)$. Figure 5c shows an FAC structure in which the CP in Figure 5b is divided into three sCPs (i.e., *M* = 3).

## 3. Experiments

### 3.1. Data

In this study, the dataset used to train the DLN comprised 38,065 frames of ultrasound images obtained from 25 volunteers, including normal and abnormal cases. Most volunteers present normal ultrasound images: there are two persons with abnormal livers, one with gallbladder problems, and one with kidney disease. Since this is not a study for diagnosing diseases, detailed abnormal cases will not be described. The abdomen is imaged, and the dataset classified into three categories: liver, kidney, and gallbladder, labeled by expert sonographers according to the guidelines specified in Reference [28]. Approximately 400 frames per organ were obtained in cine mode from various viewing angles and locations. The abdomen images of three volunteers were used as validation and test sets. A dataset comprising 3432 frames is formed by randomly dividing the cine mode of three volunteers into frames. Half of these frames were used for validation and the other half for testing. Our study and protocol were approved by the Institutional Review Board (IRB) of the Korea Centers for Disease Control and Prevention (KCDC).

### 3.2. Embedded System

**Figure 6:**
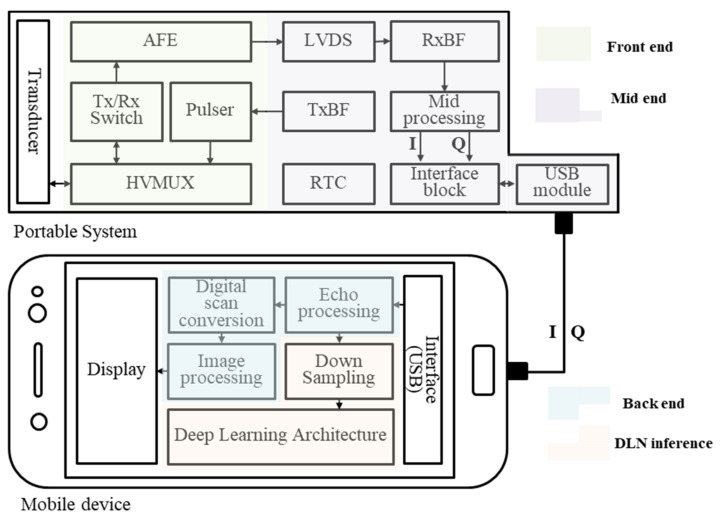
Overall block diagram of the mobile device-based portable ultrasound system for simultaneous ultrasound signal processing and deep learning network (DLN) inference.

We evaluated our method and structure in a portable ultrasound imaging system-enabled smartphone (developed by our laboratory with Hansono), which consists of a smartphone (Galaxy S7, Samsung, Korea, Android 8.0) and a 32-channel system. The portable system contains analog and digital front-ends, a mid-processor, and a USB 2.0 interface, as shown in Figure 6. Further, it has a linear array and a convex array transducer attached to the system. The mobile device contains the ultrasound image reconstruction and real-time image display on a graphical user interface with the deep learning architecture. The Galaxy S7 smartphone integrates ARM Mali-T880 GPU, which has 693.6 giga floating operations per second (GFLOPS) and 4 GB memory. In the system, we used the OpenGL ES programming model [29] to harness the computing power of the mobile GPU. We used shader storage buffer objects (SSBOs) to store the DLN parameters in OpenGL. The data in SSBOs are stored in the GPU memory until the buffer is removed. In OpenGL rendering, after one IRP is finished and initialized, the following IRP must be started. However, because SSBOs are used, there is no need to upload the DLN parameters again after initialization. The calculation results are stored in the SSBOs before starting the next IRP. After rendering the next pipeline, the calculation results stored in the SSBOs can be recalled by continuing the DLN inference.

### 3.3. Network

The deep learning model is a class of machines that learns a hierarchy of features by building high-level features from low-level ones. The CNN is a popular type of deep learning model, where trainable filters and local neighborhood pooling operations are applied in an alternating sequence, starting with raw input images. CNNs can achieve superior performance in visual object recognition and image classification tasks. CNNs have also rapidly become a methodology of choice for analyzing medical images [30], including ultrasound images. To test the method and structure proposed in this study, we explored four CNN architectures, namely, AlexNet [7], ShallowNet, MobileNet [18], and Xception [31].

AlexNet exhibits high accuracy; however, the network presented in Reference [7] is difficult to use in mobile devices due to its high computational complexity. In this work, AlexNet was used as a reference in terms of accuracy and was modified to reduce the number of nodes on the fully connected layers, from 4096 to 1024. For fast real-time computing on a mobile device, three light-weight networks (ShallowNet, MobileNet, and Xception) were chosen and evaluated. ShallowNet is a customized shallow neural network composed of two feature layers consisting of a convolution filter, max pooling, and two fully connected layers for classification. MobileNet, which is operated by a depth-wise separable convolution layer, and Xception, which uses an inception module [32], are widely adopted light-weight networks designed to reduce computational costs while maintaining high accuracy for on-device AI.

In the convolution layer, batch normalization and Rectified Linear Unit (ReLU) activation were applied after the convolution operation. In contrast, in the fully connected layer, we applied a 0.2 factor dropout and ReLU activation after the weighted sum. All CNNs used for evaluation are trained using stochastic gradient descent, which is commonly used for minimizing this cost function, where the cost over the entire training set is approximated with a cost of over 128 mini-batches of data. A learning rate of 10^−6^ ensured proper convergence for all four networks. A smaller learning rate slowed down the convergence, and a larger learning rate often caused convergence failures. The RSC and USI datasets were trained and evaluated for each network. The datasets are stored in 800 × 600 DSC format, and pre-processing must be performed to reduce their size to 64 × 64, which is used as input to the CNN. To verify the accuracy of the RSC method, the dataset of the USI region was trained separately and compared. We confirmed the performance of the proposed structure through DLN inference on a mobile device.

The parameters and calculations of the CNNs used in this study are presented in Table 1. The training process was performed in Keras with the TensorFlow framework [33] using an NVIDIA GeForce GTX 1080Ti (11 GB on-board memory) on Windows 10 for 200 optimization epochs with unit Gaussian random parameter initializations [34].

## 4. Results

We evaluate the effectiveness of building an ultrasound image training dataset using the RSC method, as well as the performance of the FAC structure that executes ultrasound image reconstruction in real-time with DLN inference. Because the FAC structure can divide the CP to operate without delay between IRPs, reconstruction processing with classification is possible on the mobile device in real time.

When calculating the network, the system calculates two-dimensional (2D) convolution in the convolution layers and a weighted sum in the fully connected layers. For minimal memory input/output (I/O) access, the area for the convolution calculation is stored in the local (shared) memory. The convolution operation is calculated in the form of a weighted sum, and the calculation of fully connected layers is processed through the reduction process using local memory.

### 4.1. RSC without FOV Dependency

The performance of the four networks was evaluated according to how ultrasonic images were built into a training dataset. Each of the four different networks were trained on the USI and RSC datasets and trained to predict three different organs. Table 2 shows the accuracy of each network that classifies abdominal ultrasound images, including the liver, gallbladder, and kidney. AC, SE and SP of USI and those of RSC were compared and the higher values were marked in bold. To quantitatively evaluate the RSC method, the accuracy, sensitivity, and specificity were used as performance measurements, which are defined as follows.

(5) $$accuracy=\left(TP+TN\right)/(TP+FN+TN+FP),\;sensitivity=TP/\left(TP+FN\right),\;specificity=TN/\left(TN+FP\right),$$

where *TP*, *FN*, *TN*, and *FP* represent the true positive, false negative, true negative, and false positive, respectively. For example, let the ground truth be liver, if the model prediction is liver, then it is judged as positive; otherwise, it is judged as negative. The results of networks trained with the RSC training dataset used in the experiment were more accurate than those in the network trained using USI data. The USI training dataset contains less information than the RSC training dataset at the same resolution. Furthermore, the networks trained by the USI training dataset can learn the FOV of ultrasound images as a feature, and thus they are less accurate.

**Figure 7:**
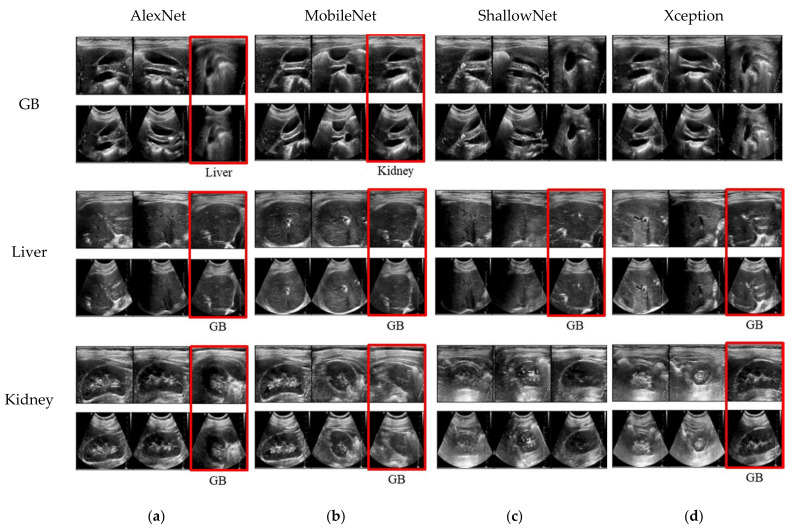
Image pairs categorized from abdomen to gallbladder, liver, and kidney. The upper row in each organ is the result obtained by the network trained by the RSC method, and the lower row is obtained by the network trained by the USI training dataset with down-sampled DSC images. Each column depicts the result of (**a**) AlexNet, (**b**) MobileNet, (**c**) ShallowNet, and (**d**) Xception. Red boxes denote errors in the results of USI training dataset, and a misclassified organ is specified below the image.

Figure 7 shows the results classified for each trained network. The rows corresponding to each class are presented as pairs of RSC and USI datasets made from the same DSC images.

**Figure 8:**
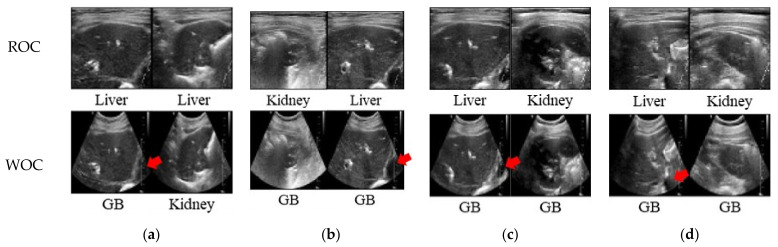
Image pairs correctly classified in the network trained by the RSC method, and misclassified in the network trained by the without cropping (WOC) method. Red arrows indicate anechoic region at deep depth, which causes misclassification. (**a**) AlexNet, (**b**) MobileNet, (**c**) ShallowNet, and (**d**) Xception.

Figure 8 shows samples of images that are trained in each network by the RSC and USI datasets, which are classified correctly and incorrectly. Of these, Figure 7 shows a pair of images that were correctly classified when training with an RSC dataset but were misclassified when training with a USI dataset.

When a feature is not clearly revealed in the image, the network trained with the USI dataset is misclassified, whereas the network trained with the RSC dataset can be classified accurately. The most common misclassified characteristic is the recognition of the liver or kidney as the gallbladder when trained with the USI dataset. The gallbladder is a pear-shaped sac, resting on the underside of the right portion of the liver [35]. In addition, the gallbladder appears anechoic, showing nothing inside the thin walls in the case of a healthy person. If a peer-shaped and anechoic structure is observed in the liver or kidney, the network trained from the USI dataset may mistake the liver or kidney as the gallbladder. This is because the amount of information varies with axial depth. Generally, there is less information at the near than at the far depth. In Figure 8, the anechoic part of the misclassified image is located under the liver. The fact that the RSC dataset has a larger FOV region than the USI dataset also affects how accurately the organs can be classified in the abdomen.

### 4.2. Structure for Real-Time Processing

To verify the proposed structure on the smartphone, after training various CNNs with the RSC dataset in a PC workstation, trained coefficients were transferred to a smartphone and used for DLN inference.

Table 3 shows how long it takes to operate DLN inference in each network when processed in the FSC structure in Figure 5b and the number of sCPs required for real-time processing in the FAC structure in Figure 5c. AlexNet has the longest processing time and the most sub-pipelines, because it has the largest number of calculations used in this study. Compared to Xception, MobileNet has a higher number of calculations, but the processing time is shorter, because the number of layers in Xception is significantly higher than that of MobileNet.

**Figure 9:**
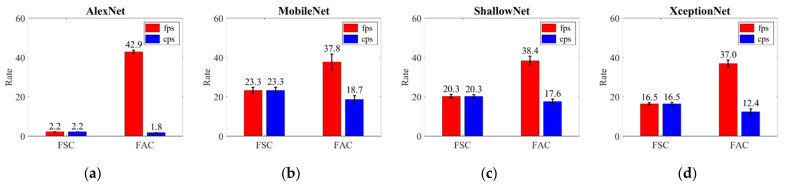
Result of ultrasound image frame rate (fps) and classification rate (cps, classification per second) during ultrasound image reconstruction with DLN inference. Frame and classification rates are represented as bar graphs with average value and variance according to frame synchronization in (**a**) AlexNet, (**b**) MobileNet, (**c**) ShallowNet, and (**d**) XceptionNet.

Figure 9 shows the results of frame and classification rates by DLN inference for classification in a mobile device with ultrasound image reconstruction. With little reduction, we can increase the frame rate for real-time ultrasound image reconstruction with DLN inference in the FAC structure rather than FSC. The larger the quantity of calculations in the CNN, the more sub-pipelines we must divide, such that the growth rate of the frame rate also increases. It is effective to increase the frame rate even when the classification rate decreases. This is because ultrasound image reconstruction must be processed in real time, rather than DLN inference.

In Figure 9, the variance of the result of the FAC structure is usually larger than that of the FSC structure. This is because we did not divide all networks equally. When there are *N* sCPs, the sum of the calculation times at each sub-pipeline is not exactly the same as the entire CP operating time. After the first sCP operation of the *N* sCP, the network is terminated at an appropriate point in the spare time. Even if a time limit offered by users is given, it is impossible to quit the running task and run other tasks when calling the OpenGL Shader. Although the following rendering must be called during the operation, it may not be immediately run, and a delay may occur.

To reduce the variance of the operation time, the most obvious method is to analyze the performance time for each shader and divide the network accurately. It is necessary to run only the task that ends before the subsequent rendering operation. However, if the runtime is strictly limited to ensure the following rendering task, the network will have to be divided into smaller pipelines, and the idle interval of the GPU will increase. This process is not effective for executing ultrasound image reconstruction using DLN inference. Each Shader operation was also designed to maximize the occupancy, such as loading the adjacent area of the image to the GPU shared memory for memory access order and efficiency of operation. Therefore, the variance of the result is inevitable, because it is intended to ensure the efficiency of the DLN inference and ultrasonic image reconstruction.

## 5. Discussions and Conclusions

We proposed an RSC method to build a training dataset for accurate training of the CNN with ultrasound images and a structure to perform ultrasound image reconstruction with DLN inference in real time on a mobile device. To evaluate the proposed RSC method, we compared the accuracy of the CNNs trained with the RSC and USI training datasets. The average accuracy of the CNNs trained with the training dataset generated by the RSC method was approximately 3% higher than that of the training dataset of the USI region. Furthermore, the proposed structure was evaluated on a portable ultrasound device and smartphone, and the frame rate was improved by 77%.

However, we think the proposed method has some limitations. First, the proposed method was validated using a mobile device-based ultrasound scanner that is equipped with only a one-array transducer and provides a fixed FOV shape. RSC is not required for devices that support multi-FOVs with one array, such as Butterfly IQ. Second, although both the RSC process for training and the down-sampling process for DLN inference employ bilinear interpolation, the pre-DSC image obtained by down-sampling the IQ signal data for the DLN inference and the $I_{RSC}$ used in the training process are different. This is because the geometry of the $I_{RSC}$ is changed during the DSC and RSC processes. Therefore, further work is necessary to investigate the effect of such differences on the inference accuracy and how to improve the performance. Finally, we designed networks that classify the three major organs of the abdomen, but we have not included a category for ‘unsure’ or ‘nothing’ images, in which three organs are not included nor accurately classified. For practical applications, the proposed method should be improved to deal with these two categories.

The RSC method of building a training dataset may improve the accuracy of various applications in training using ultrasound images. Further, the real-time structure has the advantage that ultrasound image reconstruction with DLN inference can be made without reducing the frame rate of ultrasound imaging. Using the proposed method and structure in this study, a guide for unskilled people not familiar with ultrasound imaging is effectively provided through improved POCUS.

## Figures and Tables

**Table 1:** Number of parameters and calculations in each network.

	Parameters	Calculations
AlexNet	7.1 M	305.3 M
ShallowNet	2.1 M	22.2 M
MobileNet	3.2 M	44.3 M
Xception	16.0 M	25.7 M

**Table 2:** Comparison of classification accuracies according to methods of building training data.

	USI			RSC		
	AC	SE	SP	AC	SE	SP
AlexNet	93.90	94.29	96.99	**98.55**	**95.94**	**97.90**
MobileNet	62.72	51.99	76.54	**68.73**	**55.56**	**78.72**
ShallowNet	84.00	50.65	76.42	**94.17**	**73.62**	**87.47**
Xception	88.96	67.42	**84.34**	**93.69**	**74.33**	84.32
Average	85.53	66.09	83.57	**88.79**	**74.86**	**87.10**

AC, accuracy; SE, sensitivity; SP, specificity.

**Table 3:** Processing time with ultrasound back-end process in each network and the number of dividing for real-time processing.

		Processing Time (ms)	#Dividing
CP	AlexNet	447	22
	MobileNet	43	2
	ShallowNet	49	2
	Xception	61	3
IRP		4	(not divided)

## Data Availability

Data sharing not applicable.
